# The effect of preoperative melatonin patches on sleep quality in patients undergoing urological surgery

**DOI:** 10.1590/1806-9282.20251486

**Published:** 2026-05-11

**Authors:** Çağla Toprak, Naile Akinci, Selin Ayten Akok

**Affiliations:** 1 Istanbul Atlas University, Faculty of Health Sciences, Department of Nursing – Istanbul, Turkey.; 2 Fenerbahce University, Faculty of Health Sciences, Department of Nursing – Istanbul, Turkey.; 3 Bezmialem University Hospital, Department of Urology – Istanbul, Turkey.

**Keywords:** Melatonin, Well-being, Sleep quality, Urological surgery.

## Abstract

**OBJECTIVE:**

The aim of this study was to determine the effects of transdermal melatonin patches on sleep quality in patients undergoing urological surgery.

**METHODS:**

This study is a randomized controlled trial. Fifty-seven patients hospitalized in the urology department of a university hospital in Istanbul were included. Patients were randomly assigned to two groups (melatonin and control). Patients in the melatonin group received transdermal melatonin containing 7 mg of melatonin at 11:00 PM in a quiet, darkened room the night before surgery.

**RESULTS:**

According to the results of this study, Richards-Campbell Sleep Scale sleep-quality scores increased significantly in the melatonin group (p<0.001). The control group showed lower sleep-quality scores. In addition, a statistically significant decrease was observed between the visual analog scale well-being scores before and after the application in the melatonin group (p<0.001).

**CONCLUSION:**

In this study, our results showed that melatonin patches improved sleep quality and psychological well-being in patients undergoing urological surgery.

## INTRODUCTION

In patients undergoing urological surgery (radical prostatectomy, radical cystectomy, nephrectomy, etc.), insomnia is a problem in the preoperative period due to many factors such as hospital stay, stress, anesthesia, and fear of death^1,2,3,4^. The literature shows that poor sleep quality can lead to delayed wound healing and an increased risk of postoperative infection^5,6^.

Melatonin is an important hormone that regulates both sleep and circadian rhythm^7^. According to recent meta-analyses, melatonin has been shown to improve postoperative outcomes by reducing postoperative pain, anxiety, and sleep disturbances^8,9^. Insomnia is managed in patients using pharmacological methods^10^. Tranquilisers and anti-anxiety drugs are frequently used. These drugs cause adverse side effects such as respiratory and cardiac problems, drowsiness in the patient, and prolonged recovery time. However, melatonin does not have any significant negative side effects such as respiratory depression^10,11^. It is seen in the literature that melatonin is used in many different patient groups. It has been determined that melatonin improves sleep quality in patients with total hip arthroplasty, prostatectomy, laparoscopic cholecystectomy, and breast cancer^12,13,14,15^. Melatonin is safe and does not cause addiction^16,17^. To overcome the limitations of rapid-release oral melatonin, easily applied, non-invasive transdermal melatonin patches that provide controlled release have been used. A limited number of studies in the literature address the effect of transdermal melatonin patches on postoperative sleep disorders. This study aimed to investigate the efficacy of melatonin skin patches on sleep in patients undergoing urological surgery by applying it to patients the night before surgery.

### Hypotheses of the study

H_0_: Melatonin patches have no effect on the sleep quality of patients undergoing urological surgery.

H_1_: Melatonin patches affect sleep quality of patients undergoing urological surgery.

H_2_: Melatonin patches improve well-being in patients undergoing urological surgery.

## METHODS

### Study design

It was registered in the Clinical Trials Database under protocol number NCT06910345. It was conducted according to the current CONSORT guideline, which complies with the Consolidated Standards for Clinical Trials.

### Study setting and sampling

**Figure 1:**
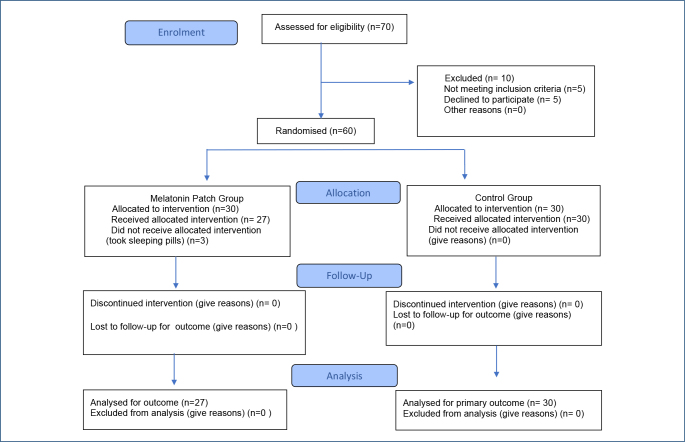
CONSORT flow diagram.

This study was conducted between 20.11.2024 and 20.03.2025 in the urological surgery ward of a Foundation University Hospital in Turkey. The sample consisted of patients who underwent urological surgery during this period and met the inclusion criteria. Sample size calculation was performed using the G*Power version 3.1.9.7 package program. Based on a similar study in the literature by Borazan et al., the effect size of the difference between sleep scores was calculated as 0.673. The power analysis determined that the sample should include at least 27 participants in each group to exceed 80% power, 5% significance, and 0.673 effect size (df=56; t=1.673)^13^. Considering the possibility of data loss, 30 patients were included for each group. Three patients in the experimental group were excluded from the study because they were prescribed additional sleeping pills. This study was completed with 57 patients: 27 in the melatonin group and 30 in the control group (Figure 1).

### Inclusion and exclusion criteria

The inclusion criteria for this study included patients who underwent urological surgery under general anesthesia, were 18 years of age or older, hemodynamically stable, and agreed to participate in the study. Patients who used hypnotic or sleeping pills during the study had delirium or other psychiatric illnesses, had hearing or speech impairment, had extensive wound/skin lesions such as drug-induced bullae or skin peeling, and patients who wanted to withdraw from the study were excluded.

### Randomization and blinding

Randomization was performed using a computer-generated random number sequence to avoid selection bias. Thirty patients were allocated to each of the two study groups. Allocation concealment was ensured through the use of sealed, opaque, and sequentially numbered envelopes, which were prepared by a researcher not involved in patient recruitment, data collection, or analysis. After patient enrollment, the next envelope in sequence was opened by the attending research nurse to determine the group assignment.

This study employed a double-masked design: both patients and outcome assessors were blinded to group allocation. Patients were accommodated in single rooms and were not informed whether they belonged to the experimental or control group, thereby minimizing expectation bias. Data collection and outcome assessment were conducted by an independent researcher who was unaware of group assignments. Participants verbally reported their responses to each item, and the assessor recorded them exactly as expressed, without interpretation, suggestion, or prompting.

### Outcomes of the study

The primary outcome of this study is that melatonin patches would improve sleep quality in patients undergoing urological surgery. The secondary outcome is that improved sleep quality affects the well-being status of the patients.

### Data collection tools

In order to collect the necessary data in the study, patient identifying characteristics form and the Richards-Campbell Sleep Scale prepared by the researchers in the light of literature information were used.

### Patients’ information form

The forms prepared by the researcher according to the literature information consist of questions such as age, height, weight, body mass index (BMI), gender, educational status, presence of chronic disease, and continuous medication use^3^.

### Richards-Campbell Sleep Scale

It was developed by Richards. Its validity and reliability study in Turkey was conducted by Karaman Özlü and Özer and was found to be suitable for the Turkish society^18^. In this study, the Cronbach-α internal consistency coefficient was found to be 0.89.

### Visual analog scale

The VAS is a 100 mm vertical or horizontal straight line in routine use. The participant is asked to rate the measured variable by marking a point between 0 and 10 on the line reflecting his/her current state, and the score is calculated by measuring the distance from the reference end of the line (point 0) to the marked point^19^. A VAS assessed patients’ well-being. Data were evaluated on a 100 mm VAS scale (0—very poor, 100—excellent). The VAS has been widely used in previous studies to assess subjective well-being, comfort, and anxiety^14^.

### Interventions

The study sample consisted of the melatonin group and the control group. Patients who met the inclusion criteria were informed about the study the evening before the operation and were assigned to the groups by obtaining their signed written consent. Afterward, the patient’s identifying characteristics form, the Richards-Campbell Sleep Scale, and VAS were filled in both groups. The same investigator carried out these procedures. Patients in both groups maintained similar environmental conditions (temperature, lighting, and visiting hours) and resided in single rooms.

### Melatonin patch group

Patients were given a melatonin sleep patch from Respro Labs^TM^ containing 7 mg melatonin at 23:00 in a quiet, dark room before surgery. The dose and brand of melatonin used in the study are similar to those reported in previous studies^17^. The melatonin sleep patch was placed on the forearm, shoulder, or lower abdomen according to the patient’s preference by a nurse who was not involved in the study. The transdermal application of melatonin aims to ensure absorption through the skin. Hairy areas may reduce skin contact, prevent adhesion, and reduce absorption efficiency, so the hairless area was chosen. If patients wish to have the patch removed during the night, they are informed to ask the clinic nurse for help. Thus, patients were not exposed to additional risks for adverse events when participating in this study. No other medication for sleep was given to the patient. Melatonin patches were left in place for 8 h. When the patient was fully awake after removing the melatonin patch at 07:00 am, the Richards-Campbell Sleep Scale and VAS were completed before the patient went to the operating theatre. No adverse events were observed during the study.

### Control group

Patients in this group received no intervention other than routine care (monitoring vital signs and daily activities). They were provided a quiet, dark room to sleep in at 23:00, as was the case in the melatonin patch group.

### Evaluation of data

SPSS (Statistical Package for the Social Sciences) version 25.0 (IBM Corp., Armonk, NY, USA) program was used for statistical analysis to evaluate the data obtained in the study. The distribution of the scores obtained from each continuous variable was analyzed using Shapiro-Wilk and Kolmogorov-Smirnov tests. An independent sample t-test was used to compare the continuous variables with normal distribution, while the Mann-Whitney U test was used for non-normal distribution. Pearson chi-square test was used to compare binary categorical variables. The Wilcoxon test was used to analyze within-group changes. Results were evaluated at a 95%CI, and significance was evaluated at p<0.05.

## RESULTS

The mean age of the participants was 51.41±17.33 years in the melatonin group and 50.40±16.83 years in the control group (Table 1).

**Table 1:** Sociodemographic characteristics of the patients.

	Melatonin		Control		p-value
	Mean±SD		Mean±SD		
Age	51.41±17.33		50.40±16.83		0.987^m^
BMI (kg/m^2^)	27.15±5.15		26.80±2.73		0.755^t^
	**n**	**%**	**n**	**%**	
Sex					
Women	6	22.22%	6	20.0%	0.837^x^2^^
Men	21	77.78%	24	80.0%	
Marital status					
Married	18	66.67%	13	43.3%	0.077^x^2^^
Single	9	33.33%	17	56.7%	
Education level					
Primary	12	44.44%	6	20.0%	**0.047** ^x^2^^
High school and above	15	55.56%	24	80.0%	
Chronic disease					
Hypertension	3	11.11%	4	13.3%	0.249^x^2^^
Heart disease	4	14.81%	9	30.0%	
Diabetes mellitus	4	14.81%	7	23.3%	
No chronic disease	16	59.26%	10	33.3%	
Smoking					
No	17	62.96%	13	43.3%	0.138^x^2^^
Yes	10	37.04%	17	56.7%	
Surgery type					
Varicocelectomy	5	18.52%	10	33.3%	0.634^x^2^^
Transurethral resection of the prostate	11	40.74%	9	30.0%	
Nephrectomy	6	22.22%	6	20.0%	
Radical prostatectomy	5	18.52%	5	16.7%	
Surgery experience					
No	10	37.04%	3	10.0%	**0.015** ^x^2^^
Yes	17	62.96%	27	90.0%	

BMI: body mass index;

^x^2^^Pearson’s chi-square test; SD: standard deviation;

^m^Mann-Whitney U test;

^t^t-test. Bold values indicate statistically significant results (p<0.05).

In the measurement after melatonin administration, mean VAS well-being scores were 16.85±10.67 in the melatonin group and 29.00±12.96 in the control group. In the measurements made after melatonin application, the mean RCSS score was found to be 69.33±23.33 in the melatonin group and 30.60±11.59 in the control group. The score was significantly higher in the melatonin group compared to the control group (p<0.001). As a result of the analysis, there was a statistically significant difference between the RCSS scores before and after the application in the melatonin group (p<0.001) (Table 2).

**Table 2:** Comparison of changes in visual analog scale, Richards-Campbell Sleep Scale scores.

	Melatonin			Control			p-value
	Mean±SD			Mean±SD			
VAS before	38.15	±	20.39	37.33	±	16.17	0.858^m^
VAS after	16.85	±	10.67	29.00	±	12.96	**<0.001** ^m^
p-value within group	**<0.001** ^w^			**<0.001** ^w^			
Sleep depth before	37.78	±	15.59	33.67	±	12.79	0.440^m^
Sleep depth after	65.37	±	26.85	31.5	±	13.59	<0.001^t^
p-value within group	**<0.001** ^w^			**0.079** ^w^			
Sleep onset latency before	35.74	±	15.42	31.17	±	14.06	0.247^t^
Sleep onset latency after	60.56	±	28.53	31.00	±	14.04	**<0.001** ^t^
p-value within group	**<0.001** ^w^			0.564^w^			
Awakenings before	41.11	±	14.16	33.83	±	14.36	0.080^m^
Awakenings after	80.00	±	27.17	32.50	±	13.76	**<0.001** ^m^
p-value within group	**<0.001** ^w^			0.161^w^			
Returning to sleep before	38.89	±	13.40	30.83	±	12.32	**0.025** ^m^
Returning to sleep after	71.85	±	26.72	31.17	±	12.64	**<0.001** ^m^
p-value within group	**<0.001** ^w^			0.608^w^			
Overall sleep quality before	37.78	±	12.73	29.33	±	12.37	**0.019** ^m^
Overall sleep quality after	68.70	±	27.41	29.83	±	12.63	**<0.001** ^m^
p-value within group	**<0.001** ^w^			0.571^w^			
RCSS sleep score before	38.26	±	12.72	31.07	±	11.50	**0.029** ^t^
RCSS sleep score after	69.33	±	23.33	30.60	±	11.59	**<0.001** ^m^
p-value within group	**<0.001** ^w^			0.215^w^			

RCSS: Richards-Campbell Sleep Scale; SD: standard deviation; VAS: visual analog scale;

^m^Mann-Whitney U test;

^t^independent sample t-test;

^w^Wilcoxon test. Bold values indicate statistically significant results (p<0.05).

According to the results of regression analysis, education level (B=-11.85, standard error [SE]=7.45, β=-0.21, p=0.118) and previous surgical experience (B=-13.32, SE=8.25, β=-0.213, p=0.112) were not found to be statistically significant in the model predicting RCSS scores. These findings indicate that no variable had a significant effect on RCSS, and baseline group differences did not significantly affect the results (Table 3). In the model predicting VAS scores, education level was found to be a significant predictor (B=8.40, SE=3.66, β=0.296, p=0.025). Accordingly, higher education levels appear to be associated with higher VAS scores. Surgical experience had no significant effect on the VAS (B=1.71, SE=4.23, β=0.054, p=0.687) (Table 3).

**Table 3:** Results of multiple linear regression analysis of the effect of educational status and surgical experience on post-procedure sleep scale and visual analog scale scores.

Scale	Factors	Unstandardized coefficients		Standardized coefficients		
		B	SE	Β	t	p
RCSS	Education level	-11.85	7.454	-0.21	-1.59	0.118
	Surgical experience	-13.322	8.252	-0.213	-1.614	0.112
	**Factors**	**B**	**SE**	**Β**	**t**	**p**
VAS	Education level	8.397	3.656	0.296	2.297	**0.025**
	Surgical experience	1.713	4.234	0.054	0.405	0.687

*p<0.05, multiple linear regression (Method=Enter). SE: standard error; RCSS: Richards-Campbell Sleep Scale; VAS: visual analog scale. Significance level: *p<0.05. Bold values indicate statistically significant results (p<0.05).

Overall, multiple regression analysis revealed that education level, one of the baseline imbalances, only affected the VAS score, while previous surgical experience was not a significant predictor of either the RCSS or VAS results (Table 3). Preoperative scores did not differ significantly between the groups (melatonin: 38.15±20.39; control: 37.33±16.17; p=0.858; Cohen’s d=0.04) Postoperative scores were significantly lower in the melatonin group (melatonin: 16.85±10.67; control: 29.00±12.96; Mann-Whitney U, p<0.001; Cohen’s d=1.01) (Table 4).

**Table 4:** Comparison of visual analog scale and Richards-Campbell Sleep Scale scores between groups with effect sizes.

Variable	Melatonin group Mean±SD	Control group Mean±SD	Test	p	Cohen’s d
VAS before	38.15±20.39	37.33±16.17	M	0.858	0.04
VAS after	16.85±10.67	29.00±12.96	M	<0.001	1.01
RCSS before total	38.26±12.72	31.07±11.50	T	0.029	0.59
RCSS after total	69.33±23.33	30.60±11.59	M	<0.001	2.07

M: Mann-Whitney U test; T: independent sample t-test; SD: standard deviation; RCSS: Richards-Campbell Sleep Scale; VAS: visual analog scale; Cohen’s d=effect size.

RCSS Sleep Scores: Preoperative scores were moderately higher in the melatonin group (melatonin: 38.26±12.72; control: 31.07±11.50; t-test, p=0.029; Cohen’s d=0.59). Postoperative scores showed a very large improvement in the melatonin group (melatonin: 69.33±23.33; control: 30.60±11.59; Mann-Whitney U, p<0.001; Cohen’s d=2.07) (Table 4).

## DISCUSSION

Melatonin, a nocturnal sleep-inducing hormone, has been extensively investigated in numerous studies and has been found to effectively regulate both sleep onset and sleep maintenance^10,20,21,22,23,24^.

In the literature, no comprehensive study has evaluated the effects of melatonin patches on psychological well-being and sleep quality in patients undergoing urological surgery. In this study, we evaluated the effects of transdermal melatonin patches on sleep quality and mental health the night before surgery.

In a study conducted by Aeschbach et al., transdermal melatonin was administered to healthy individuals experiencing jet lag, and it was found that transdermal melatonin was effective in maintaining and improving sleep, with advantages over oral melatonin^17^. In a similar sample group, a study evaluating the effects of melatonin on sleep in patients with prostate cancer reported that melatonin improved sleep disturbances^24,25^. In another study, patients who underwent elective prostatectomy received 6 mg of oral melatonin on the night before surgery and 1 h before the procedure, and postoperative sleep quality was found to improve^13^. Melatonin has been shown to improve postoperative sleep quality in various surgical interventions such as total hip arthroplasty^12^, laparoscopic cholecystectomy^14^, and breast cancer^15^. In the study, it was found that a transdermal melatonin patch containing 7 mg melatonin applied the night before surgery significantly improved sleep quality in the experimental group compared to the control group. This result is consistent with the literature, which demonstrates that transdermal melatonin is an effective method for improving sleep quality and has the advantages of no side effects, easy application, and controlled release. In contrast to the findings of this study, 10 mg of melatonin was administered before and on days 1, 2, and 3 after Transurethral Prostate Resection surgery, a urological surgical procedure. A placebo group without melatonin was compared to the melatonin-administered group, and no statistically significant effect was found on neurocognitive improvement and sleep quality compared to placebo. This difference is thought to be due to the different oral administration of melatonin in this study^26^. However, the most important methodological limitation of this study is that the control group did not use a placebo patch. The fact that the melatonin group received a visible and tactile patch, while the control group received no physical application, may have led participants to make group predictions. This could have created both expectation bias and performance bias, potentially affecting the results. In future studies, the use of placebo patches that provide complete blindness would contribute to a more reliable assessment of the true impact of the intervention. Insomnia may result from impaired psychological well-being conversely, insomnia can further deteriorate psychological well-being. All of these can contribute to anxiety, depression, and pain in patients after surgical intervention. Melatonin positively affects pain and anxiety^13^. This study evaluated the effects of melatonin patches on psychological well-being and found significantly higher levels of psychological well-being compared to the control group. Melatonin, an endogenous neurohormone secreted by the pineal gland, has multiple physiological effects that are of increasing clinical value during the surgical process. Primarily as a chronobiotic agent, it plays a critical role in synchronizing the circadian rhythm via the suprachiasmatic nucleus. Increased endogenous melatonin secretion during darkness supports the regulation of the sleep-wake cycle, offering a therapeutic opportunity in the management of circadian irregularities and sleep disorders frequently encountered in the perioperative period^7^. The use of transdermal melatonin patches is attracting attention as an innovative and applicable approach in surgical nursing.

### Strengths and weaknesses

This study has several strengths. Although melatonin has been administered to many patient groups in previous research, this is the first study to examine the effect of transdermal melatonin on preoperative sleep quality in patients undergoing urological surgery.

However, the study also has important limitations that should be acknowledged. First, the control group did not receive a placebo patch, which prevented full blinding. Because the intervention involved a visible and tangible patch, participants were likely aware of their group allocation. This constitutes a major source of potential bias and should be clearly stated as the most significant limitation.

Second, the study relied entirely on subjective, patient-reported measures (RCSS and VAS). Although these tools are valid for assessing sleep perception, the absence of any objective sleep measure (e.g., actigraphy or polysomnography) limits the robustness of the findings.

The relatively small sample size and single center design also limit the generalizability of the results. Randomization does not always achieve perfect balance in small samples, so baseline differences must be taken into account in interpreting the results.

Therefore, future research should include larger, multicenter samples and incorporate postoperative follow-up assessments. Adding objective measurements and using a placebo patch to ensure full blinding would considerably strengthen future trials.

## CONCLUSION

As a result, it was observed that transdermal melatonin can be used before urological surgical intervention; since it is a natural hormone, it safely reduces arousal and does not lead to significant side effects. Studies with larger samples are recommended for more consistent results and more study power.

## Data Availability

The datasets generated and/or analyzed during the current study are available from the corresponding author upon reasonable request.
